# Stroke services in the Middle East and adjacent region: A survey of 34 hospital-based stroke services

**DOI:** 10.3389/fneur.2022.1016376

**Published:** 2022-10-28

**Authors:** Amal. M. Al Hashmi, Ashfaq Shuaib, Yahia Imam, Dareen Amr, Hani Humaidan, Firas Al Nidawi, Ahmed Sarhan, Wessam Mustafa, Wael Khalefa, Ismail Ramadan, Fritz Sumantri Usman, Elyar Sadeghi Hokmabadi, Mohammed Ghorbani, Temeem Nassir, Farid Aladham, Athari Salmeen, Raghid Kikano, Sobri Muda, Sachin Jose, Manal Al Bulushi, Badrai Sajwani, Mohammad Wasay, Qasim Bashir, Adel Al Hazzani, Waleed Khoja, Radwan Alkadere, Haytham Osman, Abbashar Hussein, Anchalee Churojana, Nadia Hammami, Atilla Ozcan Ozdemir, Semih Giray, Erdem Gurkas, Seyd Irteza Hussain, Abdul Rahman Sallam, Ossama Yassin Mansour

**Affiliations:** ^1^Central Stroke Unit, Neuroscience Directorate, Khoula Hospital, MOH, Muscat, Oman; ^2^Division of Neurology, Department of Medicine, University of Alberta, Edmonton, AB, Canada; ^3^Neuroscience Institute, Hamad General Hospital, Doha, Qatar; ^4^Stroke and Neurointervention Unit, Alexandria University School of Medicine, Alexandria, Egypt; ^5^Stroke Unit, Salmaniya Medical Complex, Al Manamah, Bahrain; ^6^King Hamad Hospital, Al Muharaq, Bahrain; ^7^Al-Hussein University Hospital, Cairo, Egypt; ^8^Department of Neurology, Mansoura University Hospital, Mansoura, Egypt; ^9^Department of Neurology, Maady Military Hospital, Cairo, Egypt; ^10^Department of Neurology, Semoha Emergency Hospital, Alexandria University, Alexandria, Egypt; ^11^Stroke Unit, Pelni General Hospital, Jakarta, Indonesia; ^12^Neuroscience Research Center, Tabriz University of Medical Science, Tabriz, Iran; ^13^Division of Vascular and Endovascular Neurosurgery Firoozgar Hospital, Tehran, Iran; ^14^Department of Internal Medicine, Maysan Cardiac Center, MOH, Musan, Iraq; ^15^Specialty Hospital, Amman, Jordan; ^16^Department of Neurology, Jaber Al Ahmad Hospital, Kuwait City, Kuwait; ^17^Lebanese American University, Head of Interventional Radiology, Beirut, Lebanon; ^18^Department of Radiology, Pengajar Hospital UPM, FPSK, Universiti Putra Malaysia, Serdang, Malaysia; ^19^Statistical Specialist, Oman Medical Specialty Board (OMSB), Muscat, Oman; ^20^Stroke Unit, Sultan Qaboos Hospital, MOH of Oman, Salalah, Oman; ^21^Stroke Unit, Sohar Hospital, MOH of Oman, Sohar, Oman; ^22^Department of Neurology, Aga Khan University, Karachi, Pakistan; ^23^Department of Neurology, Services Institute of Medical Sciences, Lahore, Pakistan; ^24^Neuroscience Center, King Faisal Specialist Hospital and Research Center, Riyadh, Saudi Arabia; ^25^Department of Neurology, Prince Sultan Military Medical City, Riyadh, Saudi Arabia; ^26^Family Care Hospital, Riyadh, Saudi Arabia; ^27^Department of Neurology, National Ribat University, Khartoum, Sudan; ^28^Department of Neurology, El Shaab Teaching Hospital, Khartoum, Sudan; ^29^Department of Radiology, Siriraj Hospital, Mahidol University, Bangkok, Thailand; ^30^National Institute Mongi Ben Hamida of Neurology, Tunis, Tunisia; ^31^Interventional Neurology and Neurocritical Care Program, Eskisehir Osmangazi University,, Eskişehir, Turkey; ^32^Gaziantep University Medical Faculty, Gaziantep, Turkey; ^33^Stroke Center, Dr. Lutfi Kirdar City Hospital, Istanbul, Turkey; ^34^Neurological Institute, Cleveland Clinic Abu Dhabi, Abu Dhabi, United Arab Emirates; ^35^Department of Neurology, University Hospital of Sana'a, Sanan, Yemen; ^36^Department of Neurology, Faculty of Medicine, Alexandria University, Alexandria, Egypt

**Keywords:** stroke care, MENA+ region, MENA-SINO, stroke units, stroke centers

## Abstract

**Background:**

Acute stroke care is complex and requires multidisciplinary networking. There are insufficient data on stroke care in the Middle East and adjacent regions in Asia and Africa.

**Objective:**

Evaluate the state of readiness of stroke programs in the Middle East North Africa and surrounding regions (MENA+) to treat acute stroke.

**Method:**

Online questionnaire survey on the evaluation of stroke care across hospitals of MENA+ region between April 2021 and January 2022.

**Results:**

The survey was completed by 34/50 (68%) hospitals. The median population serviced by participating hospitals was 2 million. The median admission of patients with stroke/year was 600 (250–1,100). The median length of stay at the stroke units was 5 days. 34/34 (100%) of these hospitals have 24/7 CT head available. 17/34 (50%) have emergency guidelines for prehospital acute stroke care. Mechanical thrombectomy with/without IVT was available in 24/34 (70.6%). 51% was the median (IQR; 15–75%) of patients treated with IVT within 60 min from arrival. Thirty-five minutes were the median time to reverse warfarin-associated ICH.

**Conclusion:**

This is the first large study on the availability of resources for the management of acute stroke in the MENA+ region. We noted the disparity in stroke care between high-income and low-income countries. Concerted efforts are required to improve stroke care in low-income countries. Accreditation of stroke programs in the region will be helpful.

## Background

Stroke remains a leading cause of death and disability worldwide (1). Successful prevention and treatment strategies over the last five decades in high-income countries (HICs) have led to a progressive overall decline in mortality resulting in stroke becoming the fifth leading cause of death (2). Unfortunately, similar successes have not been evident in low to upper-middle-income countries (LMICs) where stroke still remains the second most common cause of death (3). Some data suggest that the burden of stroke is increasing in the Middle East and North Africa (MENA) region (3, 4). The MENA region has an estimated population of 411 million (5). A study published in 2010 reported variable stroke incidence rates in the MENA (6). The stroke incidence was 29.8 per 100,000 in Saudi Arabia and 57 per 100,000 people in Bahrain. Furthermore, the 28-day case mortality rates also varied among the MENA countries, ranging from 10% in Kuwait to 31.5% in Iran. Although the rates of strokes are comparable with those reported in high-income countries (HICs), the population of the region is much younger and therefore represents a higher burden. Another study published in 2017 (7) reported incidence rates between 16/100,000 in a prospective population based in Iran and 162/100,000 in Libya. Age-adjusted prevalence was available only from Tunisia at 184/100,000. Mortality for all strokes from the eight countries reported 30-day case fatality ranged from 9.3% in Qatar to 30% in Pakistan.

The MENA-SINO comprises stroke experts from 19 MENA and adjacent (+) regional countries (Bahrain, Egypt, Iran, Indonesia, Iraq, Jordan, Kuwait, Lebanon, Malaysia, Oman, Pakistan, Saudi Arabia, Sudan, Turkey, Thailand, United Arab Emirates (UAE), Yemen, and Qatar).

The main objective of the MENA-SINO+ is to improve education, research, and healthcare in the regional countries. The MENA-SINO organization has regular regional conferences, educational seminars, and exchanges of local and international stroke expert faculty to regional hospitals. In addition, it conducted locally relevant research and guidelines (8–11).

Acute stroke care is complex and requires multidisciplinary networking. Undoubtfully, dedicated efforts made by the regional stroke experts in the last few years have led to improvement in stroke care in the MENA region. Further improvement in stroke care will require the creation of an integrated regional stroke system at the local hospital level and across the MENA region. The lack of data on the readiness of acute stroke care from the MENA and the surrounding region (MENA+) was the driving force for this study.

## Objectives

This study aimed to assess the readiness of stroke programs in the Middle East, North Africa, and neighboring areas (MENA+) to manage acute stroke.

## Method

### The survey

An online survey composed of open and multiple-choice questions aimed at evaluating the hospital demographics, interest in stroke program certification, design of stroke program infrastructure, availability of complementary services, diagnostic capabilities, patient monitoring capabilities, availability of standard operating procedures (SOPs), and existence of effective legislation. The inter-hospital integrated protocol and the availability of various modalities for the treatment of acute stroke were also evaluated.

The survey questionnaire was available as online Supplementary material. The online survey tool (survey monkey, Palo Alto, California, USA; www.surveymonkey.com) was utilized. No compensation was offered to participants, and respondents were limited to a single response per center. The survey included general questions to identify the engagement of participants in acute stroke management and more specific questions related to the availability of facilities for thrombolysis and thrombectomy. If the participant was not actively involved in acute stroke treatment, the survey was terminated automatically.

This survey commenced in April 2021 and ended in January 2022. After 5 months the site was closed, and the data were extracted. Finally, all the survey data were fed into an excel sheet by an assigned key person to secure homogeneity, then it was double check and reviewed by the principal investigator to ensure the validity of the data.

### Participants

The survey was distributed through secure internal membership emails of the society, or postings on MENA-SINO web page and distributed to stroke and neuro interventionalists' centers' leaders (stroke program directors, stroke neurologists, neuro interventionalists, neurosurgeons, and neuro-radiologists) affiliated with the Middle East North Africa Stroke and Interventional Neurotherapies Organization (MENA-SINO). The MENA-SINO steering committee approved the study project and the questionnaire. The aim was to have at least one respondent from each of the countries comprising MENA+. Initially, 50 stroke expert affiliated hospitals showed interest to be part of the survey. However, out of the 50 initial responses, only 34 (68%) completed the survey (Appendices 1, 2).

### Statistical analysis of the data

Data were fed to the computer and analyzed using IBM SPSS software package version 28.0. (Armonk, NY: IBM Corp). Numerical data were summarized using the median and interquartile range, whereas categorical data were summarized using the frequency and percentage. Data were subsequently summarized in tables (12).

## Result

### Demographic data

Bahrain, Egypt, Iran, Indonesia, Iraq, Jordan, Kuwait, Lebanon, Malaysia, Oman, Pakistan, Sudan, Saudi Arabia, Qatar, Thailand, Turkey, Tunisia, United Arab Emirates (UAE), and Yemen took part in the survey. A total of 34 hospitals participated in the survey. 32/34 (94.1%) hospitals were interested in getting their stroke unit certified. The majority of hospitals that responded to the survey were university-based hospitals (19/34 [55.9%]). The remainder of the hospitals were associated with the ministry of health (7/34 [20.6%]), private (5/34 [14.7%]), or military (3/34 [8.8%]). The location of the stroke units/wards in the majority of hospitals was within the neurology department (18/34 [52.9%]) or within the internal medicine departments (9/34 [26.5%]). In the remainder of hospitals, the units were located within the intensive care unit, neurosurgery department, or emergency department, or other areas in the hospitals (7/34 [20.6%]).

### Structures

The median catchment population for participating hospitals was 2 million (IQR) (1.0 million−3.05 million). The number of stroke admissions to these hospitals ranged from 36 cases at Family Care Hospital in Riyadh to as high as 10,000 cases at Al-Hussein University Hospital located in Cairo. The median number of patients with stroke admitted per year at these hospitals was 600/year ([IQR] [range: 250–1,100]). The median number of admission to the stroke units/ year was 300/year ([IQR] [range: 77.5–600]).

The median length of stay at these hospitals was 8 days (IQR [range: 7–10]), whereas the median length of stay at the stroke unit was 5 days (IQR [range 5–7]). The median number of the monitored stroke unit beds at the participating hospitals was 8 (IQR [range: 4–11]) vs. 4 (IQR [range: 0–12]) for non-monitored beds (Table 1).

**Table 1:** General structures of the surveyed hospitals.

**Variable**	**Median (IQR)**	**Minimum–Maximum**
Catchment area population	2 million (1 million−3.05 million)	100,000–25 million
# of stroke admission in hospital per year	600 (250–1,100)	36–10,000
# of stroke admission to stroke unit per year	300 (77.5–600)	12–1,600
Total Hospital beds	450 (217.5–1,160)	42–2,500
# of monitored stroke unit beds	8 (4–11)	0–24
# of non-monitored stroke unit beds	4 (0–12)	0–90
Total length stroke patients stay at hospital (in days)	8 (7–10)	2–21
% of patients spending 24-72 hours in stroke unit	50.5% (30–80.75%)	5–100
% of patients spending >72 hours in stroke unit	50% (20–70%)	3–100

### Service availabilities

#### Imaging and monitoring

Head CT scanning is present at all 34 participating hospitals 24 h per day and 7 days per week. Cerebral CT angiogram facilities are delivered 24 h per day and 7 days per week at (26/34 [76.5%]) of the participating hospitals. However, brain MRI service is available only at (17/34 [50%]) of the hospitals. Clinical/biochemical emergency laboratory tests are done 24-hours per day and 7 days per week at all sites (34/34 [100%]) (Table 2).

**Table 2:** Neuroimaging and monitoring services at surveyed hospitals.

**Service availabilities**	***n*** **(%)**
CT head 24/7	34 (100)
CT cerebral angiogram 24/7	26 (76.5)
MRI brain 24/7	17 (50.0)
MRA 24/7	15 (44.1)
Neurovascular ultrasound	22 (64.7)
Intra-arterial catheter angiography	21 (61.8)
Routine ECG 24/7	32 (94.1)
Long-term-ECG with arrhythmia/AF detection	31 (91.2)
Echocardiogram	34 (100)
Clinical/biochemical emergency available 24/7	34 (100)
Continuous BP measurement	34 (100)
Pulse oximetry	33 (97.1)
Respiratory monitoring	34 (100)
Temperature recording	34 (100)

#### Other disciplines

Neurosurgical services are found at (33/34 [97%]) of the hospitals. Although the echocardiogram facility is present at all the participating hospitals (34/34 [100%]), cardiology experts are available within the hospital only at (30/34 [88.2%]) sites. Accessibility to internal medicine services is present at (32/34 [94.1%]) of the surveyed hospitals. Radiological department with neuroradiological expertise is found at (30/34 [88.2%]) hospitals, and finally, vascular surgery expertise was only available at (27/34 [79%]) hospitals.

#### Protocols and guidelines

Stroke informational manuals are available in (27/34 [79.4 %]) hospitals. Nursing manuals were available in (24/34 [82.4%]) of the sites. Only half of these hospitals (17/34 [50%]) have emergency guidelines for prehospital acute stroke care. National Institute of health stroke scale (NIHSS) was the most common scale used to access acute stroke symptoms at these hospitals (26/34 [82.4%]). The remaining 6/34 (17.6%)] hospitals used Glasgow Coma Scale (GCS).

#### Other facilities

Clinical stroke trials were conducted in (15/34 [44.1%]) of the hospitals. Post-stroke rehabilitation services were present in (30/34 [88.2%]). Tele medical/ tele radiological link with other stroke care facilities was only available in (6/34 [17.6%]) hospitals.

### Treatment options delivered for patients with stroke

#### Intravenous thrombolysis/mechanical thrombectomy

Intravenous thrombolysis (IVT) alone was offered in (7/34 [20.6%]) hospitals. Mechanical thrombectomy with/without IVT was available in (24/34 [70.6%]) hospitals. There were only 3/34 (8.8%) where the two treatments were not available. The median (IQR) percentage of patients treated with IVT for acute ischemic stroke (AIS) whose treatment is started within or <60 min following arrival to hospital was (51% [15–75%]). The median (IQR) percentage of patients with AIS eligible for IVT who received it within the appropriate time window was (56% [15–82.3%]). The median (IQR) percentage of patients treated with IVT who have a symptomatic hemorrhagic transformation (HT) within 36 h of treatment was (6% [4–10%]). The median percentage (IQR) of patients with AIS treated with endovascular interventions who developed **HT** was (5.5% [2–10%]). The median (IQR) number of decompressive craniotomies in malignant brain infarction was 10/year (3.75–20.25). The median (IQR) number of carotid interventions (surgery/stenting) was 20 /year (5–45) across all hospitals (Table 3).

**Table 3:** Treatment options delivered for AIS.

**Variable**	***n*** **(%)**	**Minimum–Maximum**
**Stroke-relevant scales**		
GCS	6 (17.6)	–
NIHSS	28 (82.4)	–
**Treatment options available for AIS across Hospitals**		
IVT alone	7 (20.6)	–
MT with/without IVT	24 (70.6)	–
Neither	3 (8.8)	–
# of carotid interventions (surgery/stenting) Per Year, median (IQR)	20 (5–45)	(0–200)
# of decompressive craniectomies performed for malignant MCA infarction, median (IQR)	10 (3.75–20.25)	(0–50)
% of patients treated with IVT & developed an sHT within 36 hours of treatment, median (IQR)	6% (4–10%)	(0–39%)
% of AIS patients treated with MT & developed HT, median (IQR)	5.5% (2–10%)	(0–29%)
% of AIS patients eligible for IVT treatment & receive it within the appropriate time window, median (IQR)	56% (15–82.25%)	(0–100%)
% of patients treated for AIS with IVT ≤ 60 min after arrival, median (IQR)	51% (15–75%)	(0–100%)
% of patients with AIS or TIA for whom an NIHSS score was documented.	78% (32.5–96%)	0–100%
Time from arrival to start of multimodal brain vascular imaging (MRI/MRA or CT/CTA) for IS patients arriving within 6 hours from last seen well, if one of these studies done	35 min (15.5–61 min)	0–100 min

#### Other types of treatments

The survey also evaluated if the hospitals had facilities to manage subarachnoid aneurysmal hemorrhages (SAHs). The number of hospitals with adequate facilities to treat SAH was 84% (48.5–84%). Nimodipine (60 mg every 4 h or 30 mg every 2 h) was started within 24 h of diagnosis and continued until 21 days after the hemorrhage or until discharge from the hospital was also an option.

Most hospitals had facilities for the reversal of anticoagulation-related ICH. Median time from arrival to start of treatment to reverse the INR with a procoagulant preparation (e.g., fresh frozen plasma, recombinant factor VIIa, prothrombin complex concentrates) for patients with warfarin-associated intracerebral hemorrhage (ICH) and an elevated INR was 35 min (35–78).

The percentage of patients with stroke or death within 24 h of diagnostic neuro-angiography after the diagnosis of SAH was 4% [4–10% as reported in 32/34 hospitals (two hospitals did not provide the data on this) was] (Table 4).

**Table 4:** Treatment options delivered for ICH/SAH.

**Variable**	**Median (IQR)**	**Minimum–Maximum**
% of patients with confirmed aneurysmal SAH & received nimodipine treatment	84% (48.5–100%)	0–100%
(Time from arrival to start treatment to reverse the INR with a procoagulant preparation) for patients with warfarin-associated ICH & an elevated INR (INR ≥1.4).	35 min (10–78 min)	0–100
% of patients with stroke or death within 24 h of diagnostic neuroangiography.	4% (0.25–10%)	0–23%

## Discussion

This is the largest study on the availability of stroke services in a large geographical area comprising 19 countries. Our study shows the availabilities of imaging resources and stroke units at the surveyed hospitals. It also highlights a wide range of variability in treatment options for acute stroke in the courtiers included in the survey. Excellent stroke care services are observed in high-income countries with immediate access to thrombolysis and thrombectomy services. Where there has been a serious lack of availability of basic care in the management of acute stroke in low-income countries.

This survey revealed a dichotomy of stroke center representation regionally. On the one hand, tertiary fully accredited centers with a standardized level of stroke care are delivered that rival major stroke centers in the West. However, on the other hand, others centers lack the basic measures of stroke care. Therefore, these results may not accurately represent the preparedness of hospitals to serve as accredited stroke centers. However, given the scarcity of data from the region we hope, the information obtained from the current study may allow for more rigorous prospective databases and registries to capture the true burden of stroke in the region.

When compared to prior studies from Asia (13), our research reveals a welcome increase in the number of stroke experts and stroke units and facilities in the region. Unfortunately, we also noted a marked disparity in stroke treatment and stroke research productivity between high- and low-income countries (14). This was particularly evident in Africa where there is a lack of awareness of stroke risk factors and recognition of symptoms of stroke and a general lack of facilities, including stroke units and rehabilitation services.

A key observation in our survey was that most hospitals were interested in stroke certification. Such certification will allow for the improvement of care in hospitals with sub-optimal stroke care. Unfortunately, such certifications have not been developed by most governments and local professionals in the MENA+ region. Our survey offers an opportunity for MENA-SINO to initiate the process in collaboration with local organizations and local governments. Collaboration between local representatives and established international organizations will be useful. The establishment of data sharing with SITS registry has improved data collection related to stroke in the MENA region and has resulted in publications that have had regional relevance (15–20).

### Limitations

There are limitations to the study. The survey was sent to a large number of hospitals representing stroke care in high and low-income countries. The majority of the participating hospitals that completed the survey were, however, tertiary hospitals in high-income countries. This may underrepresent reporting from hospitals where stroke care is sub-optimal. Given the nature of self-reporting used in the survey, and in the absence of validation or auditing, response bias may exist. The lower rates of engagement of many hospitals, especially from low-income countries in the survey, may reflect a regional lack of research culture, a lack of protected research time, and a lack of financial incentives to support such activities. The survey respondents were physicians. The underrepresentation of multidiscipline health personnel in the survey is also another limitation. Such involvement may have provided useful information on the delivery of care from the perspective of the allied health personal.

## Conclusion

This is the first large study on the availability of resources for the management of acute stroke in the MENA+ region. Our research shows a considerable variance in stroke management in the regions surveyed. Regional and international cooperation is required to advance stroke care and begin accreditation of the region's current stroke programs. Prospective registries and databases with the engagement of stakeholders to ensure engagements of more centers in future studies might help to better understand and classify the current situation of acute stroke services available in the region.

## Data availability statement

The raw data supporting the conclusions of this article will be made available by the authors, without undue reservation.

## Author contributions

AAlH: principle investigator, data organization, analysis, and manuscript writing. ASh and YI: contribution to manuscript writing and critical review. DA: data organization. SJ: data analysis. Rest of coauthors were responsible for filling out the survey of their corresponding country and institute.

## Conflict of interest

The authors declare that the research was conducted in the absence of any commercial or financial relationships that could be construed as a potential conflict of interest.

## Publisher's note

All claims expressed in this article are solely those of the authors and do not necessarily represent those of their affiliated organizations, or those of the publisher, the editors and the reviewers. Any product that may be evaluated in this article, or claim that may be made by its manufacturer, is not guaranteed or endorsed by the publisher.

## References

[1] Feigin VL Forouzanfar MH Krishnamurthi R Mensah GA Connor M Bennett D. Global and regional burden of stroke during 1990–2010: findings from the Global Burden of Disease Study 2010. Lancet. (2014) 383:245–55. 10.1016/S0140-6736(13)61953-4 24449944 PMC4181600

[2] Mozaffarian D Benjamin EJ Go AS Arnett DK Blaha MJ Cushman M et al. American Heart Association Statistics Committee, Stroke Statistics Subcommittee. Heart disease and stroke statistics-2016 update: a report from the American Heart Association. Circulation. (2016) 133:e38–60. 10.1161/CIR.0000000000000350 26673558

[3] Top 10 causes of death, WHO, 2019. 2021 Heart Disease & Stroke Statistical Update Fact Sheet—Global Burden of Disease.

[4] Feigin VL Norrving B Mensah GA. Global burden of stroke. Circ Res. (2017) 120:439–48. 10.1161/CIRCRESAHA.116.308413 28154096

[5] Khan M Al-Rukn S Alhazzani AA Aref H Moreira T Wahlgren N. Changing the face of stroke care in the Middle East North Africa region *J Neurol Sci*. (2020) 412:116727. 10.1016/j.jns.2020.116727 32062201

[6] Jackie T Masoud M Laurie A Stephen RL. The epidemiology of stroke in the Middle East and North Africa. J Neurol Sci. (2010) 295:38–40. 10.1016/j.jns.2010.05.016 20541222

[7] Streletz LJ Mushtak A Gad H Abbasi S Dimassi DM Akhtar N et al. Epidemiology of stroke in the MENA region: a systematic review. Int J Neu Dis. (2017) 1: 10-21.35177688

[8] Al Hashmi AM Ozdemir AO Shuaib A Al-Jehani H Mansour OY Alhazzani A. Current recommendations for the management of stroke patients in the middle east in the era of COVID-19 pandemic) statement from the MENA SINO. J Stroke Cerebrovasc Dis. (2020) 29:105181. 10.1016/j.jstrokecerebrovasdis.2020.105181 33066945 PMC7375312

[9] Al-Jehani H John S Hussain SI Al Hashmi A Alhamid MA Amr D. Consensus statement of the MENA-SINO on implementing care pathway for acute neurovascular emergencies during the COVID-19 pandemic. Front Neurol. (2020) 11:928. 10.3389/fneur.2020.00928 32982938 PMC7477381

[10] Al Hashmi A von Bandemer S Shuaib A Mansour OY Wassy M Ozdemir AO. Lessons learned in stroke care during COVID-19 pandemic and preparing for future pandemics in the MENA+ region: A consensus statement from the MENA+-SINO. J Neurol Scie. (2022) 432:120060. 10.1016/j.jns.2021.120060 34864375 PMC8626147

[11] Al Hashmi AM Akhtar N Al Rukin S Gaba W Mehrpour M Shuaib A. Maintaining stroke care in the era of Covid-19: A review of practices implemented in the Gulf and Iran). Neurology. (2020) 25:545–53

[12] Simpson SH. Creating a data analysis plan: what to consider when choosing statistics for a study. Can J Hosp Pharm. (2015) 68:311–7. 10.4212/cjhp.v68i4.1471 26327705 PMC4552232

[13] Suwanwela NC Poungvarin N. Stroke burden and stroke care system in Asia. Asian Stroke Advisory Panel. (2016) 64:46–51 10.4103/0028-3886.178042 26954968

[14] Apor ADAO Pagaling GT. Espiritu AI, Jamora RDG. Stroke research disparity in southeast asia: socioeconomic factors, healthcare delivery, and stroke disease burden. J Stroke Cerebrovasc Dis. (2021) 30:105481. 33249338 10.1016/j.jstrokecerebrovasdis.2020.105481

[15] Al-Rukn S Mazya M Akhtar N Hashim H Mansouri B Faouzi B et al. Stroke in the Middle-East and North Africa: A 2-year prospective observational study of intravenous thrombolysis treatment in the region. Results from the SITS-MENA Registry. Int J Stroke. (2020) 15:980–7. 10.1177/1747493019874729 31594533

[16] Jasne AS Sucharew H Alwell K Moomaw CJ Flaherty ML Adeoye O. Stroke center certification is associated with improved guideline concordance. Am J Med Qual. (2019) 34:585–9. 10.1177/1062860619835317 30868922 PMC7476218

[17] Abd-Allah F Wasay M. Roadmap for improved stroke care: implications for global stroke guidelines and action plan Int J Stroke. (2015) 10:E52. 10.1111/ijs.12528 26094678

[18] Ringelstein EB Meckes-Ferber S Hacke W Kaste M Brainin M Leys D. European stroke facilities survey: the German and Austrian perspective. Cerebrovasc Dis. (2009) 27:138–45. 10.1159/000177922 19039217

[19] Owolabi MO Thrift AG Martins M Johnson W Pandian J Abd-Allah F. The state of stroke services across the globe: Report of World Stroke Organization-World Health Organization surveys. Int J Stroke. (2021) 16:889–901. 10.1177/17474930211019568 33988062 PMC8800855

[20] McAlister FA Majumdar SR Padwal RS Fradette M Thompson A Buck B. Case management for blood pressure and lipid level control after minor stroke: PREVENTION randomized controlled trial. CMAJ. (2014) 186:577–84) 10.1503/cmaj.140053 24733770 PMC4016053

