# Supplementary material for: Stroke services in the Middle East and adjacent region: A survey of 34 hospital-based stroke services
Source: Front Neurol. 2022 Oct 28;13:1016376. doi: 10.3389/fneur.2022.1016376 (PMC9667787; doi:10.3389/fneur.2022.1016376)
Supplement: Supplementary file 1 [file Data_Sheet_1.docx]

| Abbreviation | |
| --- | --- |
| HIC | high-income countries |
| LMIC | low to upper-middle-income countries |
| MENA | Middle East and North Africa |
| MENA+ | Middle East and North Africa and adjacent countries ( Turkey, Pakistan , Malaysia, Indonesia, Philippine, Thailand and Sudan ) |
| MENA-SINO | Middle East and North Africa Stroke and Interventional Neurotherapies Organization |
| AIS | Acute Ischemic stroke |
| GCS | Glasgow Coma Scale |
| NIHSS | National Institute of Health Stroke Scale |
| IVT | Intravenous thrombolysis |
| MT | Mechanical thrombectomy |
| HT | Hemorrhagic transformation |
| ICH | Intracerebral Hemorrhage |
| SAH | Subarachnoid Hemorrhage |

*Appendix1: Participating countries and Hospitals*

| **#** | **Hospital** | **Country** |
| --- | --- | --- |
| **1** | **Salmaniya medical complex** | **Bahrain** |
| **2** | **King Hamad university hospital** | **Bahrain** |
| **3** | **Semouha emergency Hospital** | **Egypt** |
| **4** | **Louran Hospital** | **Egypt** |
| **5** | **Maadi Military Hospital** | **Egypt** |
| **6** | **Mansoura university Hospital** | **Egypt** |
| **7** | **Al-Hussein university Hospital** | **Egypt** |
| **8** | **Pelni General Hospital** | **Indonesia** |
| **9** | **Imam Reza Training & Research Hospital** | **Iran** |
| **10** | **Firoozgar University Hospital** | **Iran** |
| **11** | **Maysan Cardiac Center** | **Iraq** |
| **12** | **Specialty Hospital** | **Jordan** |
| **13** | **Jaber Al Ahmad hospital** | **Kuwait** |
| **14** | **LAU Medical Center** | **Lebanon** |
| **15** | **Hospital Pengajar UPM** | **Malaysia** |
| **16** | **Khoula Hospital** | **Oman** |
| **17** | **Sohar Hospital** | **Oman** |
| **18** | **Sulatn Qaboos Hospital** | **Oman** |
| **19** | **Nizwa Hospital** | **Oman** |
| **20** | **Aga khan University** | **Pakistan** |
| **21** | **Lahore General Hospital** | **Pakistan** |
| **22** | **Prince Sultan Military Medical City** | **SA** |
| **23** | **Family are Hospital** | **SA** |
| **24** | **King Saud University medical city** | **SA** |
| **25** | **Ribat University Hospital** | **Sudan** |
| **26** | **El shaab teaching hospital** | **Sudan** |
| **27** | **Siririaj hospital** | **Thailand** |
| **28** | **National institute Mongi ben Hamida of Neurology** | **Tunisia** |
| **29** | **Eskisehir Osmangazi University** | **Turkey** |
| **30** | **Gaziantep University Medical Faculty** | **Turkey** |
| **31** | **Dr. Lutfi kirdar city Hospital** | **Turkey** |
| **32** | **Hamad general hospital** | **Qatar** |
| **33** | **Cleveland Clinic Abu Dhabi** | **UAE** |
| **34** | **University Hospital of Sana'a** | **Yamen** |

***Appendix2: Survey Main Component***

| ***1*** | ***Demographic data.*** | ***Hospital name*** |
| --- | --- | --- |
|  |  | ***Country*** |
|  |  | ***City*** |
| ***2*** | ***Structure of the enrolled centers*** | ***Catchment area*** |
|  |  | ***Location of stroke unit*** |
|  |  | ***Number of stroke admission /year*** |
|  |  | ***Number of TIA Admission /per*** |
|  |  | ***Number of MIMIC admission /year*** |
|  |  | ***Monitored Stroke unit beds*** |
|  |  | ***Non-Monitored Stroke unit beds*** |
| ***3*** | ***Complementary disciplines.*** | ***Neurology Department*** |
|  |  | ***Neurosurgical Department*** |
|  |  | ***Internal Medicine Department*** |
|  |  | ***ICU Department*** |
|  |  | ***Radiology Department*** |
|  |  | ***Vascular surgery*** |
|  |  | ***Interventions procedures*** |
| ***4*** | ***Diagnostic capabilities:*** | ***24/7 CT head*** |
|  |  | ***24/7 cerebral angio Gram*** |
|  |  | ***24/7 MRI/MRA brain*** |
|  |  | ***Neurovascular US*** |
|  |  | ***ECG*** |
|  |  | ***ECHO*** |
| ***5*** | ***Monitoring*** | ***24 hours Heart Rate*** |
|  |  | ***24 hours Blood Pressure*** |
|  |  | ***24 hours Temperature*** |
|  |  | ***24 hours pulse oximetry*** |
| ***6*** | ***Staffing:*** | ***Qualified Medical staff in stroke*** |
|  |  | ***Nursing*** |
|  |  | ***Rehabilitation staff*** |
| ***7*** | ***Internal organisation*** | **Presence of documentation records** |
|  |  | ***Training Sessions of stroke team*** |
|  |  | ***Availability of medical manuals in stroke units*** |
|  |  | ***Stroke scales*** |
| ***8*** | ***External organisation*** | **Collaboration with other hospitals** |
|  |  | ***Post-acute rehabilitation*** |
|  |  | ***Tele medical with other stroke facilities*** |
| ***9*** | ***Internal quality management:*** | ***Type of acute stroke treatment available*** |
